# Sarcopenia and myosteatosis are accompanied by distinct biological profiles in patients with pancreatic and periampullary adenocarcinomas

**DOI:** 10.1371/journal.pone.0196235

**Published:** 2018-05-03

**Authors:** Cynthia Stretch, Jean-Michel Aubin, Beata Mickiewicz, Derek Leugner, Tariq Al-manasra, Elizabeth Tobola, Santiago Salazar, Francis R. Sutherland, Chad G. Ball, Elijah Dixon, Hans J. Vogel, Sambasivario Damaraju, Vickie E. Baracos, Oliver F. Bathe

**Affiliations:** 1 Department of Oncology, University of Calgary, Calgary, Canada; 2 Department of Surgery, University of Calgary, Calgary, Canada; 3 Department of Biological Sciences, University of Calgary, Calgary, Canada; 4 Department of Laboratory Medicine and Pathology, University of Alberta, Edmonton, Canada; 5 Department of Oncology, University of Alberta, Edmonton, Canada; University of South Alabama Mitchell Cancer Institute, UNITED STATES

## Abstract

**Introduction:**

Pancreatic and periampullary adenocarcinomas are associated with abnormal body composition visible on CT scans, including low muscle mass (sarcopenia) and low muscle radiodensity due to fat infiltration in muscle (myosteatosis). The biological and clinical correlates to these features are poorly understood.

**Methods:**

Clinical characteristics and outcomes were studied in 123 patients who underwent pancreaticoduodenectomy for pancreatic or non-pancreatic periampullary adenocarcinoma and who had available preoperative CT scans. In a subgroup of patients with pancreatic cancer (n = 29), *rectus abdominus* muscle mRNA expression was determined by cDNA microarray and in another subgroup (n = 29) ^1^H-NMR spectroscopy and gas chromatography-mass spectrometry were used to characterize the serum metabolome.

**Results:**

Muscle mass and radiodensity were not significantly correlated. Distinct groups were identified: sarcopenia (40.7%), myosteatosis (25.2%), both (11.4%). Fat distribution differed in these groups; sarcopenia associated with lower subcutaneous adipose tissue (P<0.0001) and myosteatosis associated with greater visceral adipose tissue (P<0.0001). Sarcopenia, myosteatosis and their combined presence associated with shorter survival, Log Rank P = 0.005, P = 0.06, and P = 0.002, respectively. In muscle, transcriptomic analysis suggested increased inflammation and decreased growth in sarcopenia and disrupted oxidative phosphorylation and lipid accumulation in myosteatosis. In the circulating metabolome, metabolites consistent with muscle catabolism associated with sarcopenia. Metabolites consistent with disordered carbohydrate metabolism were identified in both sarcopenia and myosteatosis.

**Discussion:**

Muscle phenotypes differ clinically and biologically. Because these muscle phenotypes are linked to poor survival, it will be imperative to delineate their pathophysiologic mechanisms, including whether they are driven by variable tumor biology or host response.

## Introduction

Pancreatic and periampullary adenocarcinomas have a poor prognosis. The only meaningful chance for cure is resection, although recurrence occurs in the majority. One of the hallmarks of these malignancies is their profound effects on the host health, including cachexia and its attendant changes in body composition [1, 2]. Cachexia is a multifactorial syndrome defined by an ongoing loss of skeletal muscle mass (with or without fat loss) that cannot be fully reversed by conventional nutritional support and leads to progressive functional impairment [3]. Functional impairment has been associated with poor operative outcomes, especially in the elderly [4–6].

In recent years, computed tomography (CT) scans have been used to evaluate cancer-associated changes in body composition [7]. Two features in particular have garnered attention: low muscle mass (sarcopenia) and decreased muscle radiodensity. Sarcopenia is a characteristic feature of cachexia [3]. Decreased muscle radiodensity is due to myosteatosis (fatty infiltration of skeletal muscles), which is known to accompany cancer [1, 8, 9]. The associations with cancer outcomes are significant. Sarcopenia is associated with increased treatment toxicity and reduced survival in several malignancies, including pancreas cancer [10–16]. Myosteatosis is associated with a shorter overall survival after resection of various malignancies, including pancreatic cancer [14, 17–20]. Low muscle mass or radiodensity may also adversely affect operative outcomes, due to impaired wound healing, depressed immunity and inability to mobilize after surgery [16, 21–26]. The relationship between sarcopenia and myosteatosis is not well described; little is known about their co-incidence. Moreover, little is known about common and divergent biological features associated with these body composition phenotypes.

In a group of patients that have all undergone Whipple pancreaticoduodenectomy for pancreatic and periampullary adenocarcinomas, we have studied operative and oncologic outcomes as a function of muscle mass and radiodensity. The associations of these abnormalities with other anthropomorphic features and other clinical factors are described. compare transcriptional profiles in muscle, as well as blood metabolic profiles, to identify discriminating biological features in patients with decreased muscle mass and low muscle radiodensity.

## Materials and methods

### Patients

Our studies were approved by the Health Research Ethics Board of Alberta (HREBA.CC-16-0769 and HREBA.CC-16-0761). Patients included in this study provided written informed consents for use of clinical data, collection and use of biological specimens for the study described here. Patients who underwent a pancreaticoduodenectomy (Whipple procedure) for pathologically confirmed pancreatic adenocarcinoma or a non-pancreatic periampullary adenocarcinoma between 2003 and 2011 and who had pre-operative CT scans available as DICOM images were included. Demographic, clinical, laboratory and operative data were collected from paper charts and electronic health records.

### Radiographic measurements

**Fig 1:**
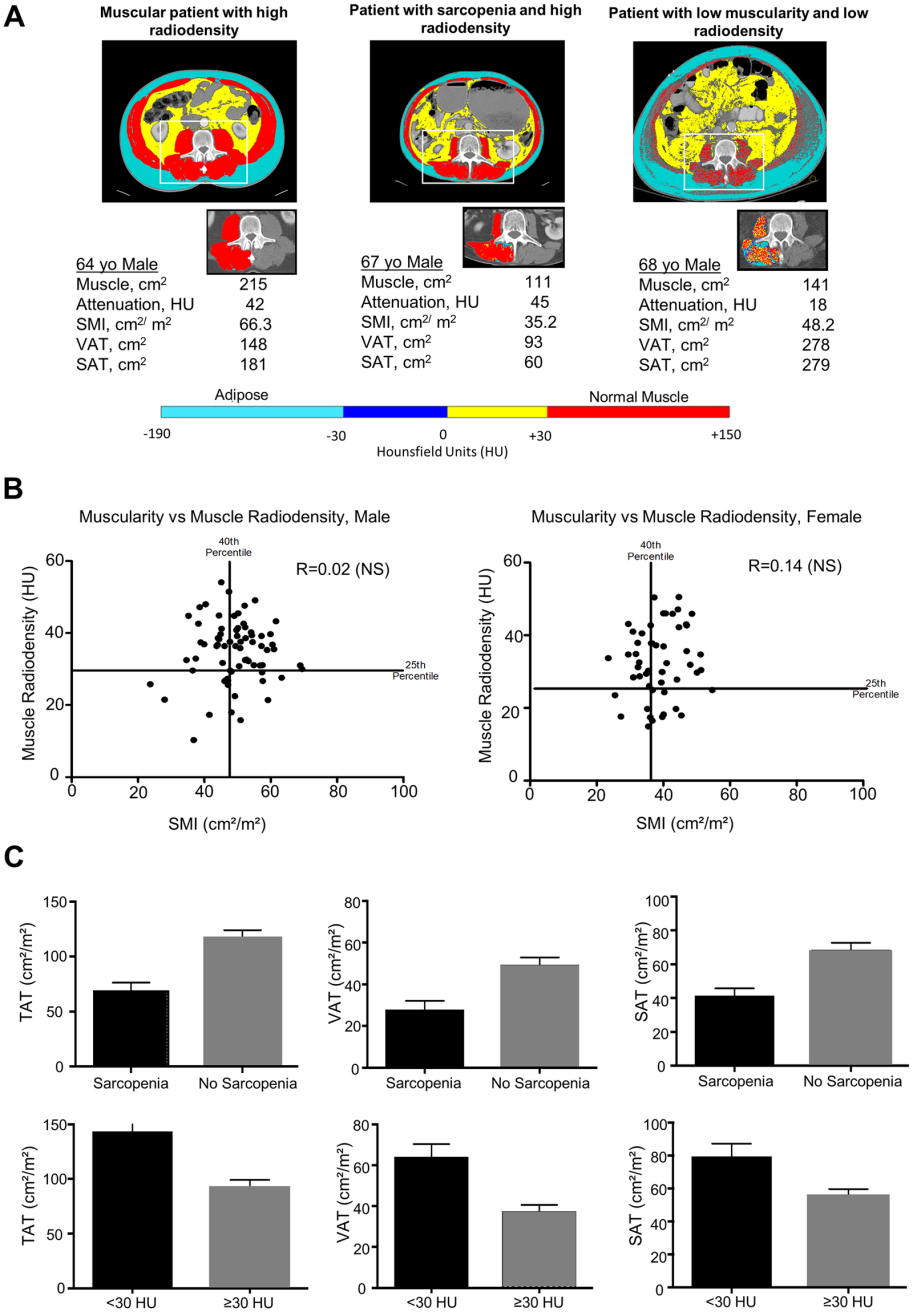
Body composition in patients with periampullary adenocarcinomas. A. Representative axial CT images from a muscular patient with high radiodensity (left panel), a patient with sarcopenia but high radiodensity (middle panel) and a patient with low muscularity and low radiodensity (right panel). The horizontal bar shows the Hounsfield Unit ranges pertaining to the isolated CT rectangles showing only the paraspinal muscles. B. Dot plot illustrating the association of muscle mass and radiodensity, in males and females. C. Differences in adipose tissue distribution in individuals with sarcopenia and low muscle radiodensity.

Preoperative digital axial CT scans done within 2 months of surgery were used to quantify skeletal muscle area as detailed in prior studies, using Slice-O-Matic software (v.4.3, Tomovision, Montreal, Canada) (Fig 1A) [7, 27]. Briefly, cross sectional surface area (cm^2^) values were calculated on images at the 3^rd^ lumbar vertebra (L3) for skeletal muscle (SM), visceral adipose tissue (VAT), subcutaneous adipose tissue (SAT) using specified Hounsfield Unit (HU) ranges: -29 to +150 HU for SM, −190 to −30 HU for SAT, and −150 to −50 HU for VAT. Total adipose tissue (TAT) was calculated by adding the surface area of the VAT and SAT. Muscle area was normalized for stature and reported as lumbar skeletal muscle index (SMI, cm^2^/m^2^). Mean muscle radiodensity was measured for the entire cross-sectional area at L3.

In addition to evaluating muscle mass as a continuous variable, we defined sarcopenia as the lowest 40^th^ percentile of SMI for each sex (<47.7 cm^2^/m^2^ for males and <36.5 cm^2^/m^2^ for females). Similarly, muscle radiodensity was analyzed as a continuous variable, and as a categorical variable (myosteatosis defined as lowest quartile of radiodensity; < 30 HU). Since muscle radiodensity was not significantly different between the sexes (S1 Fig), it was not corrected for sex. These definitions were arbitrarily based on percentile groupings, but also were similar to other studies on patients with pancreas cancer [16, 23].

### Gene expression, rectus abdominis

Rectus abdominis muscle was harvested at laparotomy. Muscles were procured at the beginning of the operation by sharp dissection, taking care not to use electrocautery, then immediately snap frozen. Methods for sample processing and microarray analysis have been previously described [28]. Microarray data have been deposited in the US National Center for Biotechnology Information Gene Expression Omnibus (GEO), series accession number GSE41726.

### Metabolomics analysis, serum

Details related to collection, storage and analysis of serum samples have been previously described [29]. Briefly, blood was procured from fasting patients with pancreatic and periampullary cancer in BD gold top vacutainers (BD Biosciences, Mississauga, ON, Canada) on the day of surgery, prior to any surgical manipulation. Samples were spun within 6 hours of collection, then frozen until analysis at -20°C. Serum samples were then analyzed by ^1^H-nuclear magnetic resonance (NMR) spectroscopy and gas chromatography-mass spectrometry (GC-MS).

### Data analysis

Descriptive statistics were used to characterize the study cohort. Continuous data were compared using t-test. Fisher’s exact test was used for comparison of categorical variables. Pearson correlation coefficients were used to test for relationships of normally distributed data. Survivals were calculated from date of surgery to date of first appearance of recurrence (for disease-free survival (DFS)) or to date of death for overall survival (OS). Survival curves were estimated using the Kaplan-Meier method, compared using the log-rank test. The effects of selected clinical factors on prognosis were analyzed using the Cox Proportional Hazard model (with bootstrap). All tests of significance were two sided and p-values < 0.05 were considered statistically significant.

In our translational studies, to limit any heterogeneity in the study group, we focused only on patients with pancreatic adenocarcinoma. In the analysis of the microarray data, the intensity values for each of the 41,000 oligonucleotide sequences was log2 transformed prior to analysis. Patients were classified according to sarcopenia and myosteatosis. Transcripts with a t-test p-values cut-off of >0.05 for each class comparison (i.e. with and without sarcopenia or with and without myosteatosis) were removed from further analysis. To select the mRNA transcripts which explain the maximum separation between defined classes, filtered transcripts were submitted to partial least squares discriminant analysis (PLS-DA). This algorithm estimates the discriminatory power of each transcript in the form of VIP values. The final list of differentially abundant transcripts included all transcripts with a VIP>1.

Differentially abundant transcripts were analyzed using QIAGEN’s Ingenuity^®^ Pathway Analysis (IPA^®^, QIAGEN Redwood City, http://www.qiagen.com/ingenuity). Biological function analysis identified the most significant functions associated with the differentially abundant genes (DAGs). Fisher’s exact test was used to calculate a p-value by IPA to determine the probability that the association between the genes and the functions is explained by chance alone. Low p-value suggests that the association between genes belonging to a function is unlikely to be due to chance. No tissue-type information was included prior to IPA analysis so as not to limit gene information available through the IPA database.

For the analysis of metabolomic data, zero values were considered as missing values. Data excluded consisted of all metabolites or features with >50% missing values, as well as unnamed or unidentified metabolites. The resulting ^1^H-NMR spectroscopy dataset contained 60 metabolites and the GC-MS dataset contained 123 metabolites for further analysis. Unsupervised principal component analysis (PCA) was conducted to identify overt outliers and any latent structures within each model. SIMCA-P+ (Version 12.0, Umetrics, Umea, Sweden) software was used for all multivariate projection modeling. Potentially significant subsets of metabolites were selected by using two-sample *t*-test assuming unequal variances (Welch’s *t*-test). Metabolites with a *p*-value < 0.30 were deemed potentially informative and were subjected to orthogonal partial least squares discriminate analyses (OPLS-DA). Compounds with variable importance in projection (VIP) ≤1 were further filtered. Metabolites were submitted to pathway analysis using MetaboAnalyst 3.4 [30] and Metscape Version 3.1.2 [31].

## Results

### Variations in body composition

123 patients were identified during the selected time period. Weight loss was reported more frequently in males (70.0 vs 51.0%, p = 0.05). As expected, males were more muscular (mean SMI 50.4 ± 13.6 vs. 38.8 ± 7.0 cm^2^/m^2^; P<0.0001), but there was no significant difference in muscle radiodensity between sexes. Men tended to have more visceral fat (55.6 ± 36.8 vs. 29.1 ± 21.5 cm^2^/m^2^; P<0.0001), and women tended to have more subcutaneous fat (78.9 ± 45.6 vs. 49.1 ± 22.8 cm^2^/m^2^; P<0.0001). Total adipose tissue was not significantly different between the sexes (S1 Fig).

Of the 123 patients, fifty patients (40.7%) were classified as having sarcopenia (regardless of myosteatosis status) and 31 patients (25.2%) were classified as having myosteatosis (regardless of sarcopenia status). Muscle mass and muscle radiodensity did not correlate well, regardless of sex (Fig 1B). Individuals with sarcopenia typically had a low BMI (Table 1). In comparison to patients with no sarcopenia, patients with sarcopenia had significantly less subcutaneous adipose tissue (44.5 ± 26.0 vs. 70.3 ± 34.8 cm^2^/m^2^; P<0.0001) and total adipose tissue (82.2 ± 47.6 vs. 122.4 ± 58.5 cm^2^/m2; P<0.0001) (Fig 1C). Visceral adipose tissue also tended to be lower in patients with sarcopenia, although this did not reach statistical significance (36.9 ± 31.7 vs. 49.6 ± 34.5 cm^2^/m^2^; p = 0.069). In comparison to patients with a normal muscle radiodensity, patients with muscle radiodensity < 30 HU had significantly greater visceral adipose tissue (65.6 ± 34.7 vs. 37.3 ± 30.6 cm^2^/m^2^; P<0.0001), subcutaneous adipose tissue (77.9 ± 37.9 vs. 53.8 ± 29.9 cm^2^/m^2^; P = 0.004) and total adipose tissue (145.5 ± 54.9 vs. 92.7 ± 52.5 cm^2^/m^2^; P<0.0001). In sum, these data demonstrate that sarcopenia and myosteatosis represent two separate and distinct clinical phenotypes.

**Table 1:** Patient characteristics as a function of low muscle mass and radiodensity. Data are expressed as mean ± SD, or as N (%).

	Muscle Mass		P	Muscle Radiodensity		P
	Sarcopenia (n = 50)	No Sarcopenia (n = 73)		< 30 HU (n = 31)	≥ 30 HU (n = 92)	
**Age (years)**	68.5 ± 10.8	66.1 ± 11.1	NS	73.5 ± 7.2	63.3 ± 10.9	<0.001
**Gender**			NS			NS
**Male**	29 (58.0%)	42 (57.5%)		18 (58.1%)	53 (57.6%)	
**Female**	21(42.0%)	31 (42.5%)		13 (41.9%)	39 (42.4%)	
**Tumor Type**			NS			NS
**Pancreatic**	37 (74.0%)	47 (64.4%)		21 (67.7%)	63 (68.5%)	
**Non-Pancreatic**	13 (26.0%)	26 (35.6%)		10 (33.3%)	29 (31.5%)	
**BMI (kg/m**^**2**^**)**	23.5 ± 3.6	26.3 ± 6.5	<0.001	25.9 ± 4.2	25.2 ± 6.3	0.061
**Symptom duration (d)**	21.0 ± 153.1	15.5 ± 187.5	NS	13.0 ± 304.9	20.5 ± 81.7	NS
**Jaundice**	43 (86.0%)	58 (79.5%)	NS	23 (74.2%)	78 (84.8%)	NS
**Gastric outlet obstruction**	43 (86.0%)	58 (79.5%)	NS	23 (74.2%)	78 (84.8%)	NS
**Charlson comorbidity Index**			NS			0.003
**≤ 2**	45 (90.0%)	65 (89.0%)		23 (74.2%)	87 (94.6%)	
**3–4**	3 (6.0%)	5 (6.9%)		4 (12.9%)	4 (4.3%)	
**≥ 5**	2 (4.0%)	3 (4.1%)		4 (12.9%)	1 (1.1%)	
**ASA Class**			NS			0.033
**1**	3 (6.0%)	3 (4.1%)		1 (3.2%)	5 (5.4%)	
**2**	23 (46.0%)	38 (52.1%)		11 (35.5%)	50 (54.3%)	
**3**	23 (46.0%)	31 (42.5%)		17 (54.8%)	37 (40.2%)	
**4**	1 (2.0%)	1 (1.4%)		2 (6.5%)	0	
**Albumin (g/L)**	34.0 ± 5.7	35.0 ± 7.6	NS	35.0 ± 6.0	34.0 ± 7.2	NS
**CEA (ug/L)**	8.2 ± 14.3	3.6 ± 5.6	0.074	7.6 ± 13.7	4.7 ± 8.6	NS
**CA 19–9 (kU/L)**	220 ± 420	1262 ± 3248	NS	2244 ± 4675	367 ± 1080	0.067
**Diabetes Mellitus**	11 (22.0%)	17 (23.3%)	NS	13 (41.9%)	15 (16.3%)	0.006

A third group based on body composition was also apparent. There were 14 patients (11.4%) who had both low muscle mass and radiodensity. These patients had features similar to what was previously described as “sarcopenic obesity” [27, 32]. In that subgroup of patients, visceral, subcutaneous and total adipose tissue were typically high (over 50^th^ percentile), yet skeletal muscle index was low. Patients in this third group had similar anthropomorphic features to each other.

Table 1 summarizes clinical characteristics as a function of muscle mass and radiodensity. We were unable to identify any clinical features that correlated with skeletal muscle index. Patients with low muscle radiodensity tended to be older, and they tended to have more significant comorbidities (higher Charlson comorbidity index, higher ASA class). Diabetes was especially associated with low muscle radiodensity.

### Operative outcomes

Operative outcomes as a function of muscle mass and radiodensity are summarized in S1 Table. Major complications (Clavien grade ≥3) occurred in 12 patients (9.8%). Three patients (2.4%) required ICU admission. Median length of stay was 14.0 days (range 6–81 days). One patient (0.8%) died as a result of the procedure. Sarcopenia did not associate with any changes in operative outcomes. Myosteatosis was associated with an increased major complication rate (P = 0.035). There was a higher anastomotic leak rate, although this did not quite reach statistical significance (P = 0.079). Length of hospitalization in patients with low muscle radiodensity tended to be longer (P = 0.053). The only operative death in this series was in a patient with myosteatosis.

### Survival

**Fig 2:**
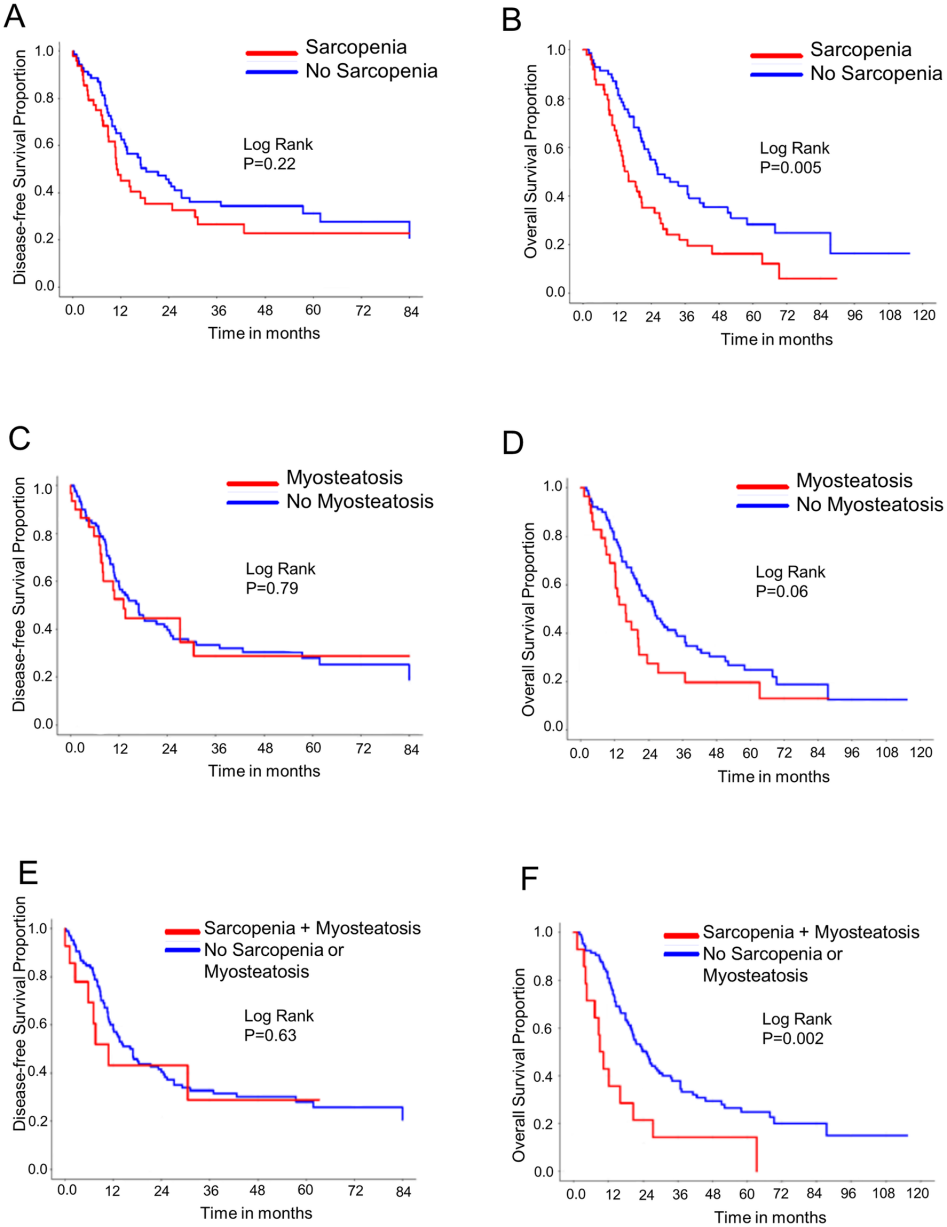
Kaplan-Meier plots. (A) Disease-free survival (DFS) as a function of sarcopenia. (B) Overall survival (OS) as a function of sarcopenia. (C) DFS as a function of myosteatosis. (D) OS as a function of myosteatosis. (E) DFS in individuals with both sarcopenia and myosteatosis. (F) OS in individuals with both sarcopenia and myosteatosis.

89 patients (72.4%) were followed until the time of death. Median follow-up for patients still alive was 44 months. Data pertaining to disease-free survival (DFS) and overall survival are summarized in Fig 2 and S2 Table.

On univariate analysis, as expected, DFS was significantly better in patients with non-pancreatic adenocarcinomas (p = 0.03, HR 0.6). CEA levels above 4.4 (p = 0.049, HR 1.7) and CA19-9 above 76.0 (p = 0.049, HR 2.1) were associated with inferior DFS. Anastomotic leaks were associated with a decreased DFS (p = 0.058, HR 0.478). DFS was not significantly linked to the presence of myosteatosis or sarcopenia. Also on univariate analysis, OS was significantly better in non-pancreatic adenocarcinomas. OS was adversely affected by infectious complications; prolonged ICU stays and high CEA levels. Median overall survival was shorter in patients with sarcopenia (26.4 vs 16.0 months, P = 0.005, HR1.8). Low muscle radiodensity as a continuous variable was significantly associated with a shorter survival (P = 0.005). Patients with myosteatosis (as we defined it) had a shorter OS, but this did not quite reach statistical significance (26.0 months vs 15.9 months, p = 0.06, HR 1.6). The coincidental presence of sarcopenia and myosteatosis was associated with a markedly reduced OS (25.2 vs 9.6 months, P = 0.002, HR 2.5).

On multivariate analysis, factors that were associated with shorter OS included an elevated CEA. Neither sarcopenia nor low muscle radiodensity was associated with a significantly reduced OS. However, the presence of both sarcopenia and myosteatosis conferred an inferior overall survival (HR 3.084 CI 1.009–9.425 P = 0.048).

### Analysis of biological samples

Low muscle mass and radiodensity appear to occur independently, represent separate phenotypes, and have additive effects on survival outcomes. To understand how these different effects on muscle arise, we evaluated the muscle transcriptome and circulating metabolome. Of the 123 patients, 29 provided muscle samples and 29 provided serum samples. Only 10 patients provided both muscle and serum and because of this, the two analyses were treated independently. Patient characteristics from samples included in the transcriptomic and metabolomic analysis are shown in S3 Table.

### Muscle transcriptome

**Fig 3:**
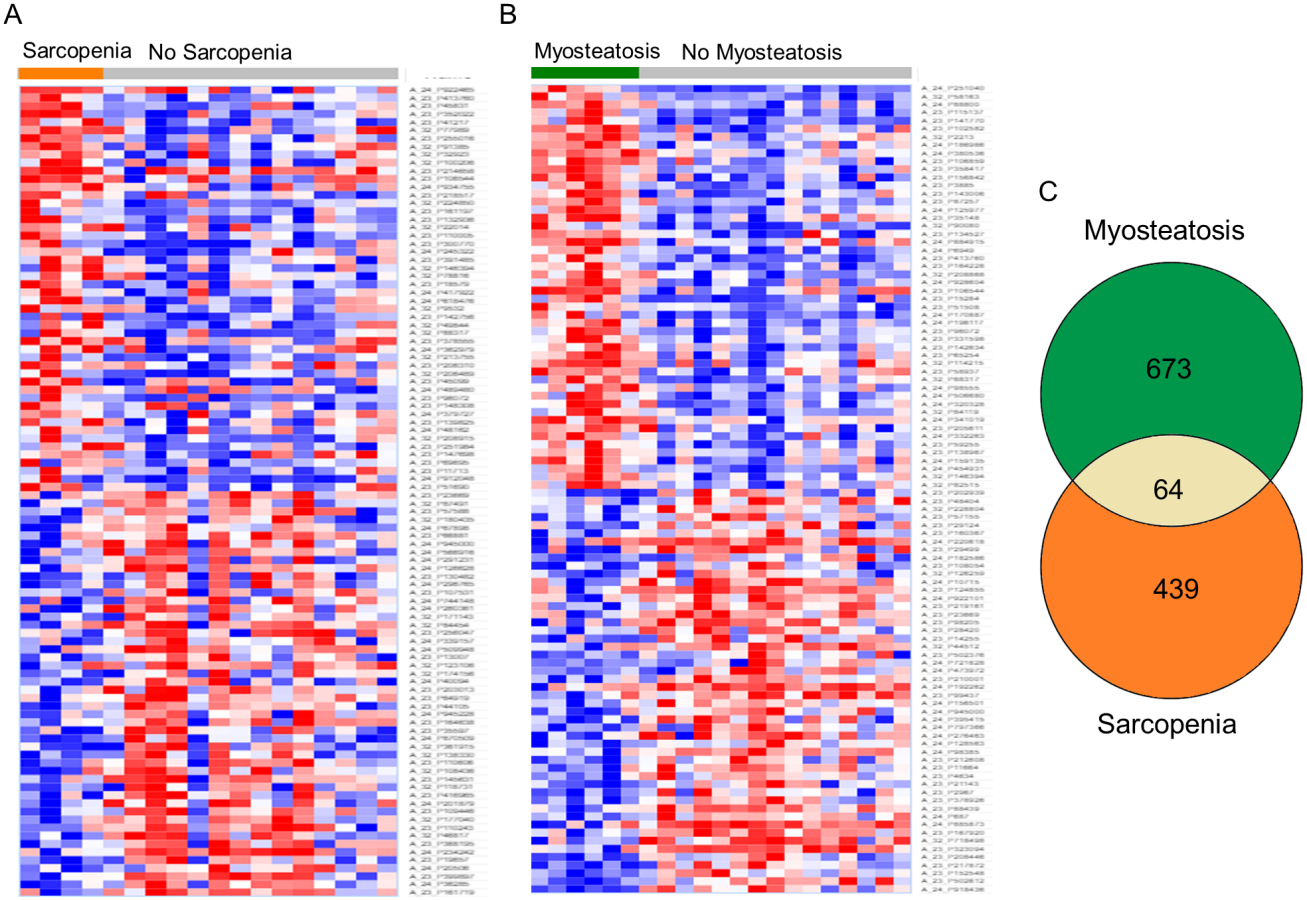
Summary of transcriptomic analysis. (A) Heatmap showing top 100 most differentially abundant transcripts (based on p-value), sarcopenia vs. normal body composition. (B) A heatmap showing top 100 most differentially abundant transcripts (based on p-value), myosteatosis vs. normal body composition. (C) Venn diagram summarizing numbers of unique and shared differentially abundant genes in sarcopenia and myosteatosis.

Transcriptomic analysis was conducted for the phenotypes of interest, sarcopenia regardless of myosteatosis status (n = 8) and myosteatosis regardless of sarcopenia status (n = 10) compared to patients with neither sarcopenia or myosteatosis (n = 15). Of 41,000 transcripts analyzed by cDNA microarray, there were 645 differentially abundant transcripts associated with sarcopenia (S4 Table). These differentially abundant transcripts mapped to 531 unique genes. When we compared patients based on presence of myosteatosis, we identified 991 differentially abundant transcripts which mapped to 772 unique genes (S5 Table). The top 100 genes associated with each phenotype are summarized in Fig 3A and 3B. Only 64 DAGs were common in the analysis of sarcopenia and myosteatosis (Fig 3C).

**Fig 4:**
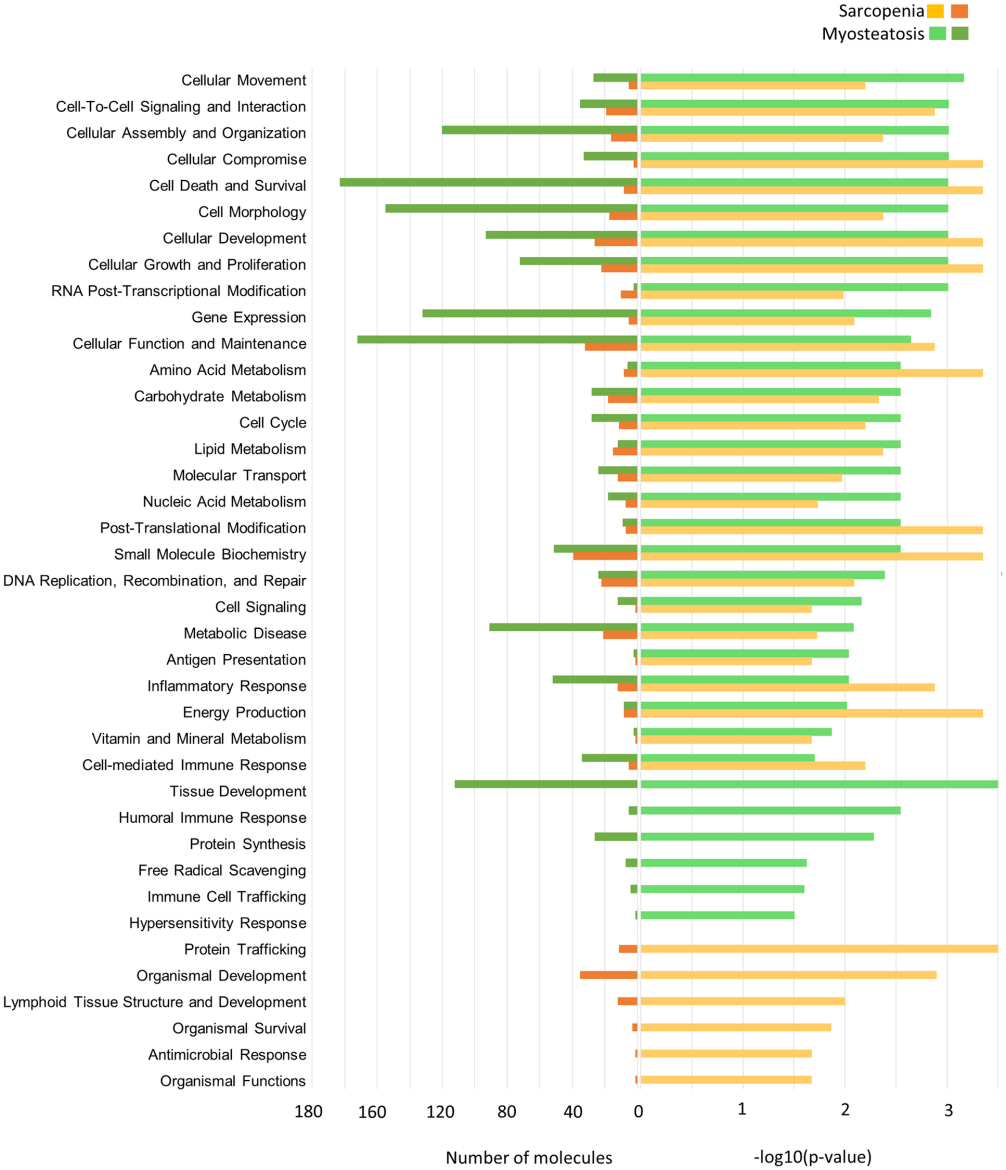
Biological functions associated with differentially abundant genes for muscle radiodensity and sarcopenia.

Biological functions associated with differentially abundant genes related to sarcopenia and myosteatosis status are summarized in Fig 4. The largest number of sarcopenia related DAGs are involved in small molecule biochemistry, cellular function and maintenance, and cellular development. Of the DAGs attributed to small molecule biochemistry and cellular function and maintenance, 13 had functional annotations related to glucose homeostasis, but based on differential abundance, it is unclear how glucose biosynthesis may be affected. DAGs involved in cellular development (e.g. PTEN, TGFBR1 and MAPK1) had lower expression in sarcopenic muscle and suggest decreased growth. The largest number of myosteatosis-related DAGs are involved in cell death and survival, cellular function and maintenance, and cell morphology. Based on the differential abundance, muscle from patients with myosteatosis are expected to have increased apoptosis, disrupted organization of cytoskeleton, and decreased branching of neurites.

The most significant canonical pathway based on sarcopenia-related DAGs was the antigen presentation pathway (Fisher’s exact test right-tailed p-value <0.0001), particularly antigen presentation to CD4+ T lymphocyte pathway. Despite lower expression of CIITA, a positive regulator of class II major histocompatibility complex (HLA) gene transcription, sarcopenic muscle had greater expression of HLA-DQA1, HLA-DQA2, HLA-DQB1, HLA-DQB2, and HLA-DQB5. Inflammation is known as an important driver in cancer associated muscle atrophy and expression of MHC class II molecules in our study support inflammation plays a role in sarcopenic muscle. Genes involved in growth pathways, typically decreased in sarcopenia, were also found to be decreased in our dataset. Genes encoding proteins involved in insulin signaling (GSK3B, MAPK1, PRKACB, PRKAR1A, PTEN, RHOQ, SYNJ1) all had lower expression in sarcopenic patients.

Oxidative phosphorylation was found to be the most significant canonical pathway (Fisher’s exact test right-tailed p-value <0.0001) based on myosteatosis-related DAGs. All 18 DAGs associated with oxidative phosphorylation had lower expression in muscles from patients with myosteatosis and encode proteins in complex I, II, IV and V of the electron transport chain. Decreased lipid oxidation likely contributes to the hallmark lipid accumulation seen in myosteatosis. Impaired oxidative phosphorylation is seen in skeletal muscles from patients with diabetes, who are also known to get myosteatosis [33]. Additionally, we identified 5 DAGs associated with lipid metabolism which may also contribute to lipid accumulation in myosteatosis (ADIPOR2 (FC = -1.39), APOL1 (FC = -1.79), APOL2 (FC = -2.10), APOO (FC = -1.90), and PON3 (FC = -1.80)).

### Circulating metabolome

In analyzing the metabolome, two complimentary platforms were employed: ^1^H-NMR spectroscopy and GC-MS. Metabolomic analysis was conducted for the phenotypes of interest, sarcopenia (regardless of myosteatosis status) and myosteatosis (regardless of sarcopenia status). However, this analysis did not result in significant a OPLS-DA model. We then further focused the analysis and compared patients with sarcopenia only (N = 8), myosteatosis only (N = 6), or neither (N = 17). The PCAs for each modality did not demonstrate any significant outliers or any latent data structures (R^2^X 0.36 for ^1^H-NMR; R^2^X 0.17 for GC-MS).

**Fig 5:**
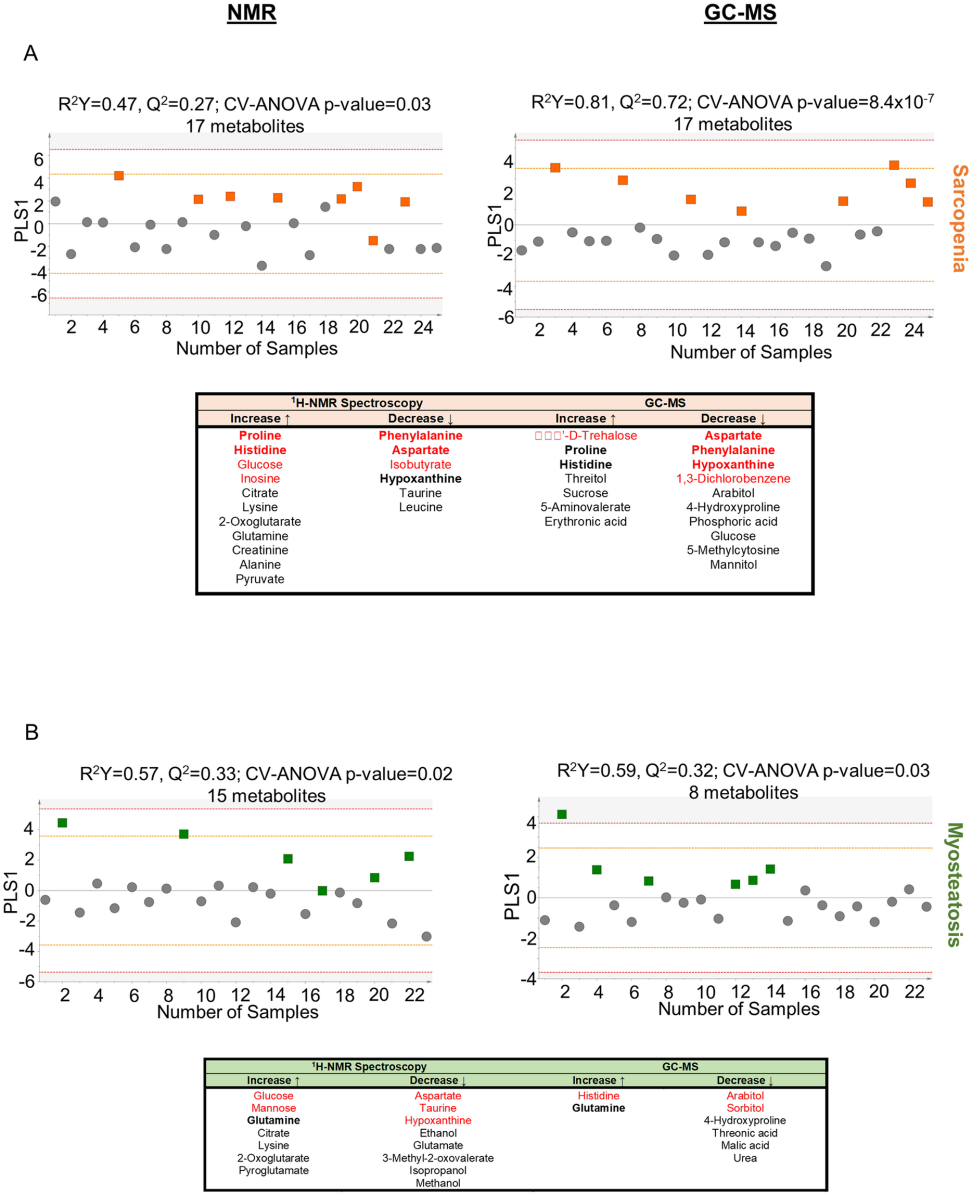
Metabolomic models that distinguish body composition phenotypes in pancreatic cancer patients. (A) OPLS-DA scores plots and metabolite lists for NMR and GC-MS metabolites: sarcopenia vs. no sarcopenia or myosteatosis. (B) OPLS-DA scores plots and metabolite lists for NMR and GC-MS metabolites: myosteatosis vs. no sarcopenia or myosteatosis. For the metabolite lists: metabolites in **bold** are shared in 1H-NMR spectroscopy and GC-MS datasets; metabolites in red have a VIP>1.

With ^1^H-NMR spectroscopy, following filtering by t-test and VIP (>1), 17 metabolites were found to distinguish sarcopenia only from no sarcopenia or myosteatosis (Fig 5A). Similarly, a 17 metabolite model could be generated from GC-MS metabolites that discriminated sarcopenia from patients with neither sarcopenia or myosteatosis. Sera from individuals with low muscle radiodensity could be distinguished from patients with neither pathological phenotype by ^1^H-NMR spectroscopy with 15 metabolites; and by GC-MS with 8 metabolites (Fig 5B).

The metabolites associated with sarcopenia are consistent with impaired glucose metabolism as suggested by the simultaneous elevation of the glucogenic amino acids histidine, proline and glutamine; this is also seen in low muscle radiodensity. Pathway analysis suggested that sarcopenia was associated with perturbed alanine, aspartate and glutamate metabolism (FDR 0.0001); histidine metabolism (FDR 0.0002); arginine and proline metabolism (FDR 0.001), as well as lysine biosynthesis (FDR 0.02). Myosteatosis was associated with alanine, aspartate and glutamate metabolism (FDR 0.04) as well as lysine metabolism (FDR 0.03). Unlike with myosteatosis, in sarcopenia, there is evidence of dysregulated urea cycle, perhaps related to protein catabolism. The increased abundance of creatinine, alanine and glutamine (present at high levels in muscle) is consistent with this.

## Discussion

Our objective was to define the relationship of sarcopenia and myosteatosis, to determine the association of each of these abnormalities with surgical and survival outcomes, and to identify whether these states have any obvious biological differences. The study was specifically designed to include patients undergoing the same procedure (Whipple procedure), where the complication rate is relatively high, so that the effects of these factors on operative outcomes would be obvious. In addition, to interrogate the underlying biology of sarcopenia and myosteatosis, transcriptomic and metabolomic studies were performed on pancreatic cancer patients.

Sarcopenia is a known feature of pancreatic cancer [1, 11, 12, 14], but there are few data on its prevalence in other periampullary adenocarcinomas. Since sarcopenia in the context of pancreatic cancer is thought to represent pathophysiologic muscle depletion, it is conceivable that recovery from surgery would be impaired, particularly if there is consequent impaired wound healing, depressed immunity and an inability to mobilize after surgery. We did not observe that sarcopenia was associated with an increased complication rate in our series. This was unexpected since several other groups have reported higher complication rates from pancreatic surgery in sarcopenic patients [12, 25, 26, 34]. We did find sarcopenia to be a poor prognostic factor in pancreatic cancer patients and this was consistent with other’s reports [11, 12, 14]. What remains unclear is whether these inferior oncologic outcomes are related to aggressive tumor biology, impaired host health, or both. In addition, it will be important to discriminate pathological sarcopenia from a premorbid ectomorphic body habitus.

Low skeletal muscle radiodensity is related to the accumulation of fat deposits within muscle [8, 9]. Our observation of worse surgical outcomes in patients with low muscle radiodensity suggests that myosteatosis reflects poor patient health. Indeed, myosteatosis occurred more frequently in individuals with more comorbidities, especially diabetes. Low muscle radiodensity was also linked to reduced OS. This was in agreement with reports from other groups [1, 16]

Our findings clearly demonstrate that sarcopenia and myosteatosis are independent abnormalities that represent two separate biological processes. They do not frequently occur co-incidentally; consequent changes in body composition differ; associated clinical factors are not the same; and they have additive effects on survival outcomes. In addition to these two abnormalities (low muscle mass and low muscle radiodensity), there was a third subgroup that had both sarcopenia and myosteatosis. Such individuals with obesity and sarcopenia have been previously described in pancreatic cancer [1, 23, 24, 32] as well as in other malignancies [27]. As demonstrated in our series, this subgroup has a particularly poor prognosis. Interestingly, diet-induced and genetically induced obesity both promote pancreatic tumor progression in mice by encouraging desmoplasia [35]. In that model, desmoplasia is encouraged by the activation of pancreatic stellate cells, which occurs with the recruitment of tumor-associated neutrophils by adipocyte-secreted IL-1β.

To gain an understanding of the biological differences between cancer-associated muscle atrophy and fat infiltration, we evaluated the muscle transcriptome and circulating metabolome. At the level of the muscle, diabetogenic-like alterations in carbohydrate and lipid metabolism were especially apparent with low muscle radiodensity. This was in keeping with the observation that low muscle radiodensity is more common with diabetes in this series as well as others [36, 37]. The diabetes associated with pancreatic cancer is unusual, as there is often an absence of obesity and there is a rapid progression to insulin dependence [38]. Insulin encourages tumor growth *in vitro*; in clinical series, the need for exogenous insulin therapy was associated with an increased risk of recurrence of colorectal cancer and hepatocellular carcinoma [39–41]. Cancer-associated muscle wasting is associated with metabolic impairments, including insulin insensitivity [42]. Our results at the muscle and serum level agree with this.

Several limitations need to be acknowledged in our current analysis. First, this study was a single centre retrospective study using data from stored muscle and serum samples. Second, the patients included in the transcriptomic study were not the same as those included in the metabolomic study. In the future, analysis of biological samples from the same patients would enhance our understanding of metabolic differences between patients with sarcopenia only, myosteatosis only or both occurring together. Third, the sample sizes for our analyses were small. Given the discovery stage of the study and data analysis from multi omics platforms, statistical power analysis was not attempted.

There is intense interest regarding the underlying pathophysiology of cancer-associated sarcopenia and low muscle radiodensity and their impact on oncologic outcomes. Our work demonstrates that these two abnormalities are clinically and biologically distinct. When they do occur coincidentally, the adverse effects on patient prognosis are particularly profound. In future work, it will be important to understand what drives the heterogeneous responses to cancer. One possibility is that both muscle abnormalities are driven by different molecular factors originating from tumor. Alternatively, there may be differences in host susceptibility. Our work contributes to a growing body of evidence linking changes of body composition with clinical outcomes in cancer. Once sufficient information is available to consider therapeutic interventions, it will become important to identify at risk phenotypes, using preoperative CT imaging or biomarkers.

## Supporting information

S1 Fig Sex-related differences in muscularity, muscle radiodensity, and adipose tissue distribution. (PDF)

S1 Table Operative outcomes as a function of sarcopenia and myosteatosis. Data are expressed as mean ± SD, or as N (%).
(PDF)

S2 Table Summary of factors associated with disease-free survival and overall survival on univariate and multivariate analysis. (PDF)

S3 Table Patient characteristics for the transcriptomic and metabolomic datasets. (PDF)

S4 Table List of differentially abundant genes associated with sarcopenia. (XLSX)

S5 Table List of differentially abundant genes associated with myosteatosis. (XLSX)
