# Supplementary material for: Sarcopenia and myosteatosis are accompanied by distinct biological profiles in patients with pancreatic and periampullary adenocarcinomas
Source: PLoS One. 2018 May 3;13(5):e0196235. doi: 10.1371/journal.pone.0196235 (PMC5933771; doi:10.1371/journal.pone.0196235)
Supplement: S1 Fig — (PDF) [file pone.0196235.s001.pdf]

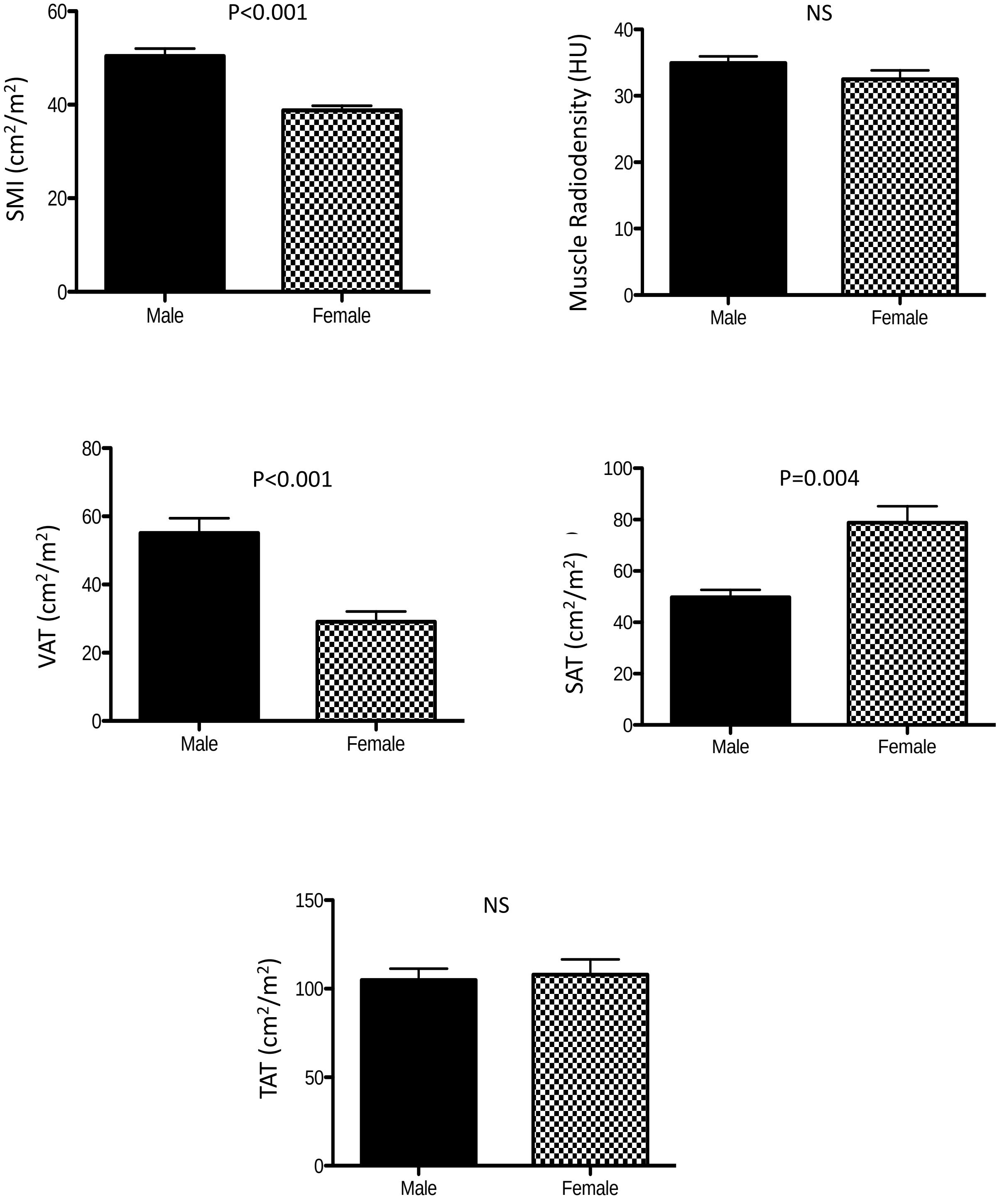

Supplementary Figure S1. Sex-related differences in muscularity, muscle attenuation, and adipose tissue distribution.
