# Supplementary material for: Sarcopenia and myosteatosis are accompanied by distinct biological profiles in patients with pancreatic and periampullary adenocarcinomas
Source: PLoS One. 2018 May 3;13(5):e0196235. doi: 10.1371/journal.pone.0196235 (PMC5933771; doi:10.1371/journal.pone.0196235)
Supplement: S1 Table — Data are expressed as mean ± SD, or as N (%). (PDF) [file pone.0196235.s002.pdf]

Supplementary Table S1. Operative outcomes as a function of sarcopenia and myosteatosis. Data are expressed as mean ± SD, or as N (%).

	**Muscle Mass**		**P**	**Muscle Attenuation**		**P**
	**Sarcopenia (n=50)**	**No Sarcopenia (n=73)**		**Low Radiodensity (n=31)**	**High Radiodensity (n=92)**	
Blood loss (mL)	534 ± 408	443 ± 282	NS	489 ± 347	477 ± 340	NS
Operative time (min)	357 ± 68	369 ± 74	NS	363 ± 57	364 ± 76	NS
ICU LOS	0.3 ± 2.1	0.1 ± 0.6	NS	0.6 ± 2.7	0.1 ± 0.5	NS
LOS (days)	16.6 ± 8.2	16.1 ± 10.5	NS	20.2 ±14.3	15.0 ± 6.9	0.053
Complications						
Clavien ≥ 3	6 (12%)	6 (8.2%)	NS	6 (19.4%)	6 (6.5%)	0.035
Delayed Gastric Emptying	16 (32%)	27 (37.0%)	NS	15 (48.4%)	28 (30.4)	NS
Anastomotic leak	9 (18%)	9 (12.3%)	NS	8 (25.8%)	10 (10.9%)	0.074
Infectious complications	12 (24%)	22 (30.1%)	NS	12 (38.7%)	22 (23.9%)	NS
