# Supplementary material for: Sarcopenia and myosteatosis are accompanied by distinct biological profiles in patients with pancreatic and periampullary adenocarcinomas
Source: PLoS One. 2018 May 3;13(5):e0196235. doi: 10.1371/journal.pone.0196235 (PMC5933771; doi:10.1371/journal.pone.0196235)
Supplement: S2 Table — (PDF) [file pone.0196235.s003.pdf]

Supplementary Table S2. Summary of factors associated with disease-free survival and overall survival on univariate and multivariate analysis.

	Univariate analysis			Multivariate analysis		
	P	HR	CI	P	HR	CI
**DFS**						
Non-pancreatic cancer	0.030	0.570	0.342 – 0.951			
CEA > 4.4	0.049	1.709	0.997 – 2.929			
CA 19-9 > 76.0	0.049	2.097	0.989 – 4.449			
Anastomotic leak	0.058	0.478	0.220 – 1.042			
**OS**						
Non-pancreatic cancer	0.001	0.421	0.254 – 0.699	0.284	0.490	0.133 – 1.804
Muscle attenuation> 27.6 HU	0.005	0.966	0.942 – 0.990			
CEA > 4.4	0.015	1.896	1.121 – 3.208	<0.001	1.071	1.031 – 1.112
ICU LOS > 2.0 d	<0.001	1.369	1.116 – 1.680			
Infectious complication	0.097	0.652	0.392 - 1.085	0.541	1.448	0.442 – 4.737
Myosteatosis	0.062	1.559	0.974 – 2.494			
Sarcopenia	0.005	1.798	1.182 – 2.735			
Sarco40 & Myo25	0.002	2.462	1.360 – 4.458	0.048	3.084	1.009 – 9.425
