# Supplementary material for: Sarcopenia and myosteatosis are accompanied by distinct biological profiles in patients with pancreatic and periampullary adenocarcinomas
Source: PLoS One. 2018 May 3;13(5):e0196235. doi: 10.1371/journal.pone.0196235 (PMC5933771; doi:10.1371/journal.pone.0196235)
Supplement: S3 Table — (PDF) [file pone.0196235.s004.pdf]

Supplementary Table S3. Summary of characteristics of samples from pancreatic cancer used for biological analysis. Data are expressed as mean ±, or as N (%)

	Serum metabolomic analysis			Skeletal muscle transcriptomic analysis		
	Sarcopenia	Myosteatosis	Neither	Sarcopenia	Myosteatosis	Neither
Number of Samples						
^{1}H-NMR Spectroscopy	8	6	17			
GC-MS	8	6	17			
cDNA Microarray				8	10	15
Age, years	68 ± 10	74 ± 4**	59 ± 11	63 ± 11	73 ± 8**	63 ± 9
Sex, male N (%)	8 (100)	1 (17)	8 (47)	4 (50)	4 (40)	8 (53)
Diabetes, N (%)	1 (17)	3 (60)	1 (8)	0 (0)	3 (30)	2 (13)
Muscle Mass (SMI; cm^2/m^2)	42 ± 5***	43 ± 7	47 ± 7	38± 8***	44 ± 8	49 ± 7
Muscle Radiodensity (HU)	38 ± 7	22 ± 5***	41 ± 7	32 ± 10	26 ± 3***	39 ± 5

Significance testing compared the “Sarcopenia” or “Myosteatosis” groups with the “Neither” group. *$P < 0.05$; **$P < 0.01$; ***$P < 0.001$
